# A school-based program implemented by community providers previously trained for the prevention of eating and weight-related problems in secondary-school adolescents: the MABIC study protocol

**DOI:** 10.1186/1471-2458-13-955

**Published:** 2013-10-12

**Authors:** David Sánchez-Carracedo, Gemma López-Guimerà, Jordi Fauquet, Juan Ramón Barrada, Montserrat Pàmias, Joaquim Puntí, Mireia Querol, Esther Trepat

**Affiliations:** 1 Department of Clinical and Health Psychology, Universitat Autònoma de Barcelona, 08193 Bellaterra (Cerdanyola del Vallès) Barcelona, Spain; 2 Research Unit on Eating and Weight-related Behaviors (UCAP), Universitat Autònoma de Barcelona, 08193 Bellaterra (Cerdanyola del Vallès) Barcelona, Spain; 3 Department of Psychobiology and Methodology of Health Sciences, Universitat Autònoma de Barcelona, 08193 Bellaterra (Cerdanyola del Vallès) Barcelona, Spain; 4 Neuroimaging Research Group, IMIM (Hospital del Mar Medical Research Institute), Barcelona Biomedical Research Park, C/ Doctor Aiguader, 88, 08003 Barcelona, Spain; 5 Department of Psychology and Sociology, University of Zaragoza, 44003 Teruel, Spain; 6 Mental Health Unit of the Parc Taulí Health Corporation (CSPT), 08208 Sabadell, Barcelona, Spain; 7 Institute of Psychology Foundation, República Argentina, 182, 08023 Barcelona, Spain

**Keywords:** Prevention, Effectiveness, Disordered eating, Eating disorders, Overweight, Obesity, Eating and weight-related problems

## Abstract

**Background:**

The prevention of eating disorders and disordered eating are increasingly recognized as public health priorities. Challenges in this field included moving from efficacy to effectiveness and developing an integrated approach to the prevention of a broad spectrum of eating and weight-related problems. A previous efficacy trial indicated that a universal disordered eating prevention program, based on the social cognitive model, media literacy educational approach and cognitive dissonance theory, reduced risk factors for disordered eating, but it is unclear whether this program has effects under more real-world conditions. The main aim of this effectiveness trial protocol is to test whether this program has effects when incorporating an integrated approach to prevention and when previously-trained community providers implement the intervention.

**Methods/design:**

The research design involved a multi-center non-randomized controlled trial with baseline, post and 1-year follow-up measures. Six schools from the city of Sabadell (close to Barcelona) participated in the intervention group, and eleven schools from four towns neighboring Sabadell participated in the control group. A total of 174 girls and 180 boys in the intervention group, and 484 girls and 490 boys in the control group were registered in class lists prior to baseline. A total of 18 community providers, secondary-school class tutors, nurses from the Catalan Government’s Health and School Program, and health promotion technicians from Sabadell City Council were trained and delivered the program. Shared risk factors of eating and weight-related problems were assessed as main measures.

**Discussion:**

It will be vital for progress in disordered eating prevention to conduct effectiveness trials, which test whether interventions are effective when delivered by community providers under ecologically valid conditions, as opposed to tightly controlled research trials. The MABIC project will provide new contributions in this transition from efficacy to effectiveness and new data about progress in the integrated approach to prevention. Pending the results, the effectiveness trial meets the effectiveness standards set down by the Society for Prevention Research. This study will provide new evidence to improve and enhance disordered eating prevention programs.

**Trial registration:**

Current Controlled Trials ISRCTN47682626

## Background

### Relevance of disordered eating prevention

The high prevalence of eating disorders and disordered eating, together with their chronic tendency, high comorbidity with other mental disorders, association with serious physical and psychosocial health consequences and resistance to available treatments, as well as the fact that most individuals with eating disorders do not receive treatment, constitute powerful reasons for an approach focused on their prevention [1].

Lifetime prevalence worldwide for eating disorders in young women (including anorexia nervosa, bulimia nervosa and eating disorders not otherwise specified / EDNOS) is estimated at about 5% [2], according to the criteria for the Diagnostic and Statistical Manual of Mental Disorders (4th ed. [DSM-IV]) [3]. Other studies report prevalence rates of about 10%, including threshold, subthreshold, and partial eating disorders [4-6]. In Spain, various epidemiological studies [7-10] report similar data, revealing a prevalence for eating disorders of 4-6% in girls and young women aged 12–21 years [11]. In fact, eating disorders are the third most prevalent chronic illness in adolescent girls after obesity and asthma [12].

The more prevalent forms, threshold and EDNOS eating disorders, are marked by chronicity, relapse, distress, functional impairment, risk of future obesity, depression, suicide attempts, anxiety disorders, substance abuse, and morbidity [13-17]. Eating disorders are associated with some of the highest mortality rates for any psychological disorder [18]. In fact, a relevant review found standardized mortality ratios of 5.9 for anorexia nervosa and 1.9 for both bulimia nervosa and EDNOS, reporting that one in 5 individuals with anorexia nervosa who died had committed suicide [19].

In addition, it is currently estimated that in the US 17% of girls and 11% of boys report binge eating episodes in the last month, 12% of girls and 3% of boys report vomiting to control their weight, and 24% of girls and 8% of boys report wanting to be thinner [20]. In Spain, research has found that approximately 28% of girls and 12% of boys are engaged in unhealthy weight-control behaviors [21], while nearly 7% of boys and 14% of girls reached the cut-off point on instruments for assessing disordered eating behaviors and attitudes [22]. The high prevalence of these attitudes and behaviors is a cause for concern, given that they have been shown to be associated with less healthy dietary patterns, and may have a negative effect on both physical and psychosocial health [23-31]. Moreover, these attitudes and behaviors increase the risk of weight gain, overweight status, disordered eating behaviors and the development of clinical eating disorders [32-37].

Finally, in relation to treatment resistance, clinical controlled trials show that 23% of patients with anorexia nervosa and 41% of patients with bulimia nervosa terminated their therapy prematurely [38]. Also, non-response to cognitive-behavioral treatment, considered the most effective to date, is at around 50%, much higher than desirable [39,40].

### Progress in disordered eating prevention research

A range of disordered eating preventive programs have been developed in recent decades, and several reviews about their effects have been carried out in different settings and with different populations [1,41-48]. Data from meta-analyses [41,45,46] show that larger effects were found for programs with interactive exercises focused on reduce specific risk factors that predict the onset of eating pathology (vs. psycho-educational programs), with multiple sessions (vs. single session), that were assessed with validated measures, that were delivered by experts (vs. by teachers), and that targeted high-risk individuals (vs. a universal population). In general, prevention programs conducted with adolescents targeting eating problems and eating disorders have led to improvements in knowledge, though only a small number of such programs have succeeded in improving significant disordered eating attitudes and behaviors from baseline scores to follow-up [43].

### New challenges: moving from efficacy to effectiveness and integrated prevention of obesity and disordered eating

Prevention scientists distinguish between efficacy trials and effectiveness trials [49,50]. *Efficacy* refers to the beneficial effects of a program or policy under optimal conditions of delivery [51], and involves the systematic and scientific evaluation of whether an intervention works.

In this type of trial, efficacy is assessed when intervention is administered under optimal conditions, in which participants are often homogeneous, and intervention is delivered by well trained and closely supervised research clinic staff [49,50]. Efficacy is one of the two dimensions established by the American Psychological Association (APA) for the evaluation of its intervention guidelines [52]. It should be stressed that program efficacy does not guarantee that the program will have effects under real-world conditions. *Effectiveness* refers to the beneficial effects of a program or policy under more real-world conditions [51]. It is the second dimension established by the APA, *clinical utility*, and refers to the applicability, feasibility and usefulness of the intervention in the local or specific setting in which it is implemented [52]. This dimension is essential for determining the generalizability to the real world of an intervention whose efficacy has been established. An important feature of effectiveness is whether the intervention has been shown to be effective when delivery is by community providers (e.g., teachers), who have many competing demands on their time and attention every day, as opposed to experts or professional researchers [53]. This feature is one of the key standards of the Society of Prevention Research in relation to establishing the effectiveness of prevention programs [51].

It is in this context that, recently, there has been concern in this field about whether programs and policies developed and tested in research settings could be implemented with predictable success in schools, social agencies, and communities [51], which would require moving from efficacy to effectiveness trials in prevention research [54]. A few reports have described programs of efficacy to effectiveness research in various fields of prevention, including substance abuse, childhood obesity, and HIV infection [55-57], but examples of this transition in disordered eating prevention fields are scarce.

To date, the majority of prevention trials in the disordered eating field have been efficacy trials, which tested whether prevention programs achieve effects under strongly controlled research conditions in which they are delivered by developers or closely supervised experts [58]. However, programs worthy of dissemination must also be effective under real-world conditions and assessed in effectiveness trials. Such effectiveness trials are uncommon in this field, but are vital to progress and future research [59]. Up to now, and to the best of our knowledge, only one targeted prevention program, The Body Project, whose efficacy had been previously evaluated [60], has also been assessed in an effectiveness trial [61]. Subsequent effectiveness trials have showed that the Body Project retains its effectiveness even when it is delivered in a universal format with college students from sororities [62-65]. There are indeed other universal prevention programs whose effectiveness has been evaluated when intervention is delivered by previously-trained teachers, such as the PriMa program [66-68] or the POPS program [69]. However, both programs lack previous efficacy trials and, in the case of the POPS program, evaluation of its results is still under way. Therefore, to the best of our knowledge, our study would be the first effectiveness trial in disordered eating universal prevention research with adolescents whose efficacy has been previously established.

On the other hand, empirically-supported reasons and practical considerations have led to eating and weight-related problems being seen as part of a continuum and to the development of interventions aimed at preventing this broad spectrum of problems [70]. Eating and weight-related problems include anorexic and bulimic behaviors (such as fasting, vomiting and the use of laxatives, diet pills or diuretics), unhealthy dieting practices (such as dieting skipping meals), body dissatisfaction, binge-eating disorder, overweight and obesity [71]. Reasons for this integrated approach to prevention include (i) the co-occurrence of these problems and easy progress from one problem to another over time; (ii) identification of shared risk factors; (iii) a possible lack of coherence in the messages being transmitted in the field of obesity and eating disorders prevention; and (iv) the efficiency of implementing programs aimed at preventing a broad spectrum of eating and weight-related problems, rather than using separate programs [70].

In particular, special attention is being paid to shared risk factors of eating disorders and obesity [70,72-75], and calls for the development of an integrated approach to prevention in both fields are increasing [59,70,76-78].

However, to date, research in the two fields has followed quite separate paths. There is major concern over the possible harmful influence of preventive interventions in one field on the other–in particular, about the effects obesity prevention programs might have on variables such as body image, excessive weight concerns, weight-related teasing, or engaging in restrictive dieting and unhealthy weight-control behaviors. These effects could cancel out the efforts and achievements made in the field of prevention of disordered eating and body dissatisfaction [78-80]. This is a controversial issue: we do not know whether such unintended psychological harm occurs or not, as in obesity prevention programs the variables related to disordered eating and body image have been scarcely evaluated [81]. In spite of this, we have indirect evidence of this possible harmful influence in the high frequency of unhealthy weight-control behaviors among overweight and obese adolescents, situated at between 30% and 70%, depending on sex and the type of population studied [21,82,83].

Some recent obesity prevention programs have been concerned to evaluate their potential effects on these types of variables [84,85], and some programs aimed at preventing shared risk factors of obesity and eating disorders have been developed [60,86,87]. But developments in this field are still quite few and far between. As we shall see, our study also introduces some elements of this integrated approach.

### The MABIC project

Our research team have developed and assessed a universal school-based disordered eating prevention program that combines the empirically supported components most commonly associated with successful interventions of the type cited above, except for those related to targeted prevention [88]. A detailed description can be found in the published intervention manual [89]. The efficacy of the program was assessed under strong methodological conditions, including delivery conducted by two of the developers, both professional researchers (GLG and DSC). The study was registered in the International Standard Randomized Controlled Trial Number Register (ISRCTNR) of Current Controlled Trials (no. ISRCTN07896919). The program was effective in generating positive changes in eating attitudes and beauty ideal influences at 6-month follow-up, with effect sizes greater, on average, than those obtained previously in selective-universal programs, and similar to or greater than those achieved by targeted prevention programs [88].

In 2009, a new research project was launched, led by our research team in conjunction with the Parc Taulí Health Corporation (CSPT in Spanish) and the Institute of Psychology Foundation (FIP in Spanish) and supported by a grant from the Spanish Ministry of Education and Innovation (no. PSI2009-08956). The present research is part of this project, and sets out to assess the effectiveness of the aforementioned program in a new effectiveness trial. The effect of the program will be evaluated now when delivered by previously trained community providers.

In line with the integrated approach, the training of community providers in the present study was based on an integrated approach to prevention. Moreover, the main outcomes were focused on shared risk factors of eating disorders and obesity. Indeed, the origin of the project’s name, the MABIC project, is a Spanish acronym for four shared risk factors of eating disorders and obesity with empirical support: “M” for media (“Medios de comunicación” in Spanish); “A” for disordered eating (e.g., dieting) (“Alimentación alterada” in Spanish); “B” for weight-related teasing, (“Burlas relacionadas con el peso” in Spanish); and “IC” for body dissatisfaction (“Insatisfacción Corporal” in Spanish). The effectiveness trial was registered in the ISRCTNR of Current Controlled Trials (no. ISRCTN47682626). Recently, our team obtained a new grant from the Spanish Ministry of Economy and Competitiveness (no. PSI2012-31077) for the MABIC-II project, a continuation of the previous one.

### Study objective

In sum, the main aim of this study is to assess the effectiveness of a universal-selective disordered eating prevention program, whose efficacy has been demonstrated previously, in reducing shared risk factors of eating disorders and obesity. The effectiveness will be evaluated when the program is delivered by community providers previously trained in an integrated approach to the prevention of eating disorders and obesity.

## Methods/design

### Study design

The design of this study was a multi-center non-randomized controlled trial involving measurement of a cohort of adolescent girls and boys at baseline, post-test and 1-year follow-up. As stated above, the study is registered in the international register of Current Controlled Trials [90] (ISRCTN47682626).

### Ethical approval

The study was approved by the Clinical Research Ethics Committee of the CSPT (ref: 2009–512, cooperation partner of the MABIC project). Family informed consent was requested prior to the beginning of the study. Participants’ assent was also requested. The confidentiality of participating adolescents was protected with coded data, and the data processing is anonymous.

### Sample size calculation

The sample size calculation was based on a medium effect size of the primary outcomes between the intervention and control group. This effect size threshold was based on the results of the literature review about the disordered eating prevention field, reporting that the effect sizes have tended to be small on average in efficacy trials, although some programs have produced medium effect sizes for continuous outcomes, which have been considered clinically significant [1]. Moreover, effectiveness trials often obtain small effects sizes [61]. Consequently, a total sample size of 292 allows detecting medium effect sizes with a power level of 80% at a significance level of 0.05. Finally, under these assumptions and assuming that is estimated that usually between 15-20% of the participants will be lost at the follow-up [43], the final effective sample size is established at 351 participants.

### Setting, recruitment and participants

The intervention is school-based and addressed to secondary schools. Schools are widely recognized as appropriate sites for interventions to prevent disordered eating among adolescents [47]. They offer the potential for sustained interactions with young people at a developmentally appropriate age, where they are already in a learning environment [91]. Governments, schools and curricular authorities are also increasingly recognizing the potential for addressing disordered eating issues in the school setting [47,92].

The CSPT was the basic intermediary for recruiting the health community providers responsible for implementing part of the program. Sabadell (a city close to Barcelona) is the CSPT’s main area of influence. In addition, the CSPT, as the only public health care service in this area, has the ethical commitment to provide health care to the entire population, so that the possibility of participating in the group receiving the intervention should be offered to all secondary schools in the city. For these reasons, the intervention group was made up exclusively of schools located in Sabadell. In order to constitute a control group of similar socio-demographic characteristics to those of the intervention group, schools allocated to the control group were recruited from towns neighboring Sabadell. In this way, a possible “spillover” effect was also avoided [91]. Using separate control and intervention schools is a preferred strategy for many researchers, so that there are no contamination concerns (potential for young people to talk to their peers about the research), and possible demographic differences can be controlled with strategies in place to match samples for demographic characteristics, or through covariate analyses [47].

Six schools, two public (with a total of eight classes) and four grant-aided private (GAP/ with a total of six classes) from Sabadell participated in the intervention group. The total number of participants registered in class lists prior to baseline in this group was 354 (174 girls and 180 boys). Eleven schools, ten public (with a total of thirty-five classes) and one GAP (with a total of two classes) from the towns of Badia del Vallès, Cerdanyola del Vallès, Ripollet and Rubí, all neighboring Sabadell, participated in the control group. The total number of participants registered in class lists prior to baseline in this group was 974 (484 girls and 490 boys). All participants were eighth-grade (second-grade in Spanish secondary education), with a usual age range of 13–14 years. A complete description of the socio-demographic characteristics of the two groups will take into account sex, Body Mass Index (BMI), weight status, socioeconomic level and origin (ethnicity/country of birth).

These adolescents participated in assessments at three points: baseline (prior to the beginning of the program); post-class (two weeks after the final session of the program); and follow-up (one year from the beginning of the intervention). A flow chart showing response patterns (enrolment, allocation, retention rates, main attrition causes, etc.) in the intervention and control groups will be plotted across the different assessments when the data analysis is carried out.

### Description of the effectiveness trial

The trial comprises two main phases. The first phase involves the development of the training program and the training of the community providers, while the second phase consists of the implementation of the intervention and the pre, post and 1-year follow-up assessments. Data analysis of results is still pending.

### Phase 1: the MABIC training program and the Health and School Program

In 2009 we designed a pilot training program that was administered at the end of the same year to tutors and educational psychologists from two public schools in the city of Sabadell, in two different editions. These professionals implemented the preventive program in theirrespective secondary schools. After its administration, we conducted in-depth interviews with these professionals in order to make the appropriate improvements to both the structure and content of the training program.

Throughout the year 2010 three official editions of the definitive training program were scheduled for the months of January-February, July, and October-November. Table 1 shows the structure and content of this training program.

**Table 1:** Content and structure of the community provider training program

***Sessions***	***Content***
***First session*.**	• Justification of the course and presentation of the MABIC project and its objectives.
**Concept of eating and weight-related problems (EWRP)**	
	• Presentation of the objectives and content of the course.
	• Eating and weight-related problems concept (EWRP).
	• Prevalence data of EWRP.
	• Empirical reasons and practical considerations for using an integrated approach.
	• Empirically supported shared risk factors for different EWRP that are potentially modifiable via preventive initiatives:
	- Dieting and unhealthy weight-control behaviors
	- Media use and beauty ideal internalization
	- Body dissatisfaction and weight and body concerns
	- Weight-related teasing
	- Frequency of family meals
***Second session.***	• Definition and criteria of obesity and overweight in adolescents.
**Overweight, obesity and eating disorders: Prevalence, causes and consequences (first part).**	
	• Prevalence of obesity.
	• Health problems associated with obesity.
	• Changing false beliefs about the linear association between BMI and health problems through the presentation of evidence from scientific studies.
	• Psychosocial consequences of obesity: weight-related teasing and weight bias.
	• Genetic causes of obesity and the importance of obesogenic environment.
	• Importance of physical activity.
	• Failure of weight-loss diets in the treatment of obesity in the long term.
	• Importance of obesity prevention. Presentation of recent preventive initiatives in our country.
	• Definition and criteria of eating disorders (ED) and its importance.
	• Prevalence of ED.
	• Health problems associated with ED.
	• Characteristics of ED: analysis of the thoughts and behaviors characteristic of people with these problems.
***Third session.***	• Risk factors for ED.
**Eating disorders: Prevalence, causes and consequences (second part) / Effects of restrictive and fad diets.**	
	• Early detection, parents’ reaction and presentation of available health care resources.
	• Analysis of participants’ beliefs about the effects of restrictive diets.
	• Presentation of 10 empirically supported reasons not to go on restrictive diets.
	• Effects of restrictive and fad diets and correction of false beliefs about it.
	• Data about determinants of weight and about the difficulty of voluntary weight modification in the long term.
	• Presentation of the thrifty genotype theory.
	• Brief demonstration of the administration of the “Effects of diets” section from the preventive program that should be administered to participants.
***Fourth session.***	• Presentation of the new *Health at Every Size* paradigm in the treatment of obesity.
**Nutrition, eating habits and Media Literacy.**	
	• Presentation of a positive approach to nutrition education and activities for working with adolescents and families from this perspective.
	• Brief demonstration of the administration of the “Nutrition” component of the preventive program that should be administered to participants.
	• Prevalence data about media use and supported data about the influence of the media in the development of EWRP.
	• What Media Literacy is and is not.
	• Presentation of the “Action learning” model and the four necessary steps (Awareness / Analysis / Reflection / Action) for developing media literacy skills.
	• Using media literacy and cognitive dissonance techniques to reduce the internalization of the thinness and beauty ideals.
	• Brief demonstration of the administration of the “Media Literacy” component of the preventive program that should be administered to participants.
***Fifth session*.**	• Presentation of the structure of the preventive program.
**The preventive program: “Eating, the feminine beauty ideal and the media. How to train secondary-school students to be critical”.**	• Role-playing: Acting out of some parts of the “Nutrition” component by health staff. Teaching use of the scripts and the manual. Reinforcement and feedback on the implementation.
	• Role-playing: Acting out of some parts of the component “Media Literacy” by school staff. Teaching use of the scripts and the manual. Reinforcement and feedback on the implementation.
	• Delivering the manual, the videos to be used as model-guide and accreditations.
	• Closure of the course.

The training program was addressed to both teachers and health care professionals from the Health and School Program and Health Promotion Service of Sabadell City Council. The Health and School Program [93] was launched in October 2004 and is an initiative of the Catalonian Government’s Department of Education and Universities and Department of Health to promote prevention and health and to improve the coordination of relevant action aimed at secondary-school students in public and GAP schools. The overall objective of this program is to improve the health of adolescents through initiatives in school settings by promoting health, the prevention of risk situations and early attention to health problems. Education in nutrition and the prevention of eating disorders is one of the main priorities. Primary care nurses participating in this program spent two hours per week in secondary schools working on these aspects. The CSPT is responsible for support and supervision in the specific areas of the Health and School Program in the city of Sabadell, and helped to facilitate the participation of nurses in the MABIC project. These health professionals would be responsible for delivering one of the components of the preventive program (Nutrition). The Health Promotion technical staff at Sabadell City Council, who collaborate with the MABIC project and with nurses from the Health and School Program, would cover for nurses who could not deliver the planned interventions for various reasons. Previously-trained teachers, the majority being secondary school class tutors, would be responsible for delivering the other component of the preventive program (Media Literacy).

The training program was provided through the training plan section of the Center for Pedagogical Resources of *Vallès Occidental I* (Sabadell), and was therefore offered to all professionals working in its area of influence. A total of 63 community providers were trained, with the following distribution: 38 secondary school class tutors from 20 schools in Sabadell, 1 clinical psychologist, 7 Health Promotion technicians from Sabadell City Council, and 17 nurses from the Health and School Program. Among this trained staff, the project enjoyed the participation of 18 community providers linked to schools recruited from Sabadell and forming part of the intervention group: 11 secondary school class tutors, 5 nurses from the Health and School Program, and 7 health promotion technicians from Sabadell City Council.

The training program schedule involved five 3-hour sessions per week (see Table 1). Training sessions were carried out by the first and second authors of this paper (DSC and GLG). Teachers were provided with all the necessary didactic material. A copy of the intervention manual was provided to all participating community providers. DSC and GLG are co-authors of the intervention program and made videos showing how they administered some components of the preventive intervention. These videos were also given to community providers participating in the project MABIC to be used as a model-guide. The training program was based entirely on the integrated approach to eating and weight-related problems. Finally, in addition to the training, the research team offered support and supervision throughout the whole process to those professionals requesting it.

The MABIC project training program was accredited by the Education Department of the Government of Catalonia for the school staff, and by the Continuing Medical Education Commission of the National Health System and the Catalan Council of Continued Training for the Health Professions for the health staff.

### Phase 2: implementation of the intervention and the assessment of its effectiveness

#### Planning and time frame of the study

From the end of 2009 and throughout 2010, in order to explain the project and to recruit schools, the director of the MABIC project held several meetings with those responsible for education and health services, as well as with the secondary school head teachers and heads of health promotion services from the participating town and city councils. Participation in the project was offered to all schools, both public and GAP, in the five localities involved, and was voluntary. The only requirement for participating in the project was making a commitment to carry out the three planned assessments. An additional requirement for participation in the intervention group was that class tutors at the participating schools would attend the training program prior to administration of the intervention. Family consent and participants’ assent were required prior to commencement of the study. Inclusion criteria for participants were provision of their consent and completion of the assessment protocol. No exclusion criteria were applied a priori, and full eighth-grade classes in the recruited schools participated. Only one school was excluded in the intervention group for having a population with special educational needs. In this school the intervention was implemented but no assessments were carried out. Participants with incomplete assessments or problems that could introduce relevant bias in the data would be excluded.

Undergraduate and Master’s students recruited by the project performed all the assessments. The assessment protocol was administered at three points in the normal class schedule, with anonymity of the responses being guaranteed. Each assessment lasted about 60 minutes.

In the baseline and follow-up assessments, small groups of 6–8 participants were led to a private room while the protocol was being administered. There, the trained staff took weight and height measures following a standardized procedure. Baseline assessments took place between January and March 2011 in both the intervention and control groups. Intervention began in each school one week after the baseline assessment, and a fidelity control of the program implementation was carried out (see Intervention section). The post assessment took place two weeks after completion of the intervention, between April and June 2011. Both assessments were carried out within the academic year 2010–11. Follow-up assessment took place in the following academic year, 2011–12, between February and March 2012.

In March 2013, participating community providers were required to complete a questionnaire for evaluating the training process and the project as a whole. A screening for possible eating disorder cases was included in the assessment protocol. Scoring above the cut-off point of the ChEAT and SCOFF-c questionnaires (see Measures section) was used as criterion. In fulfillment of the ethical commitments of every public health care service, the CSPT and those responsible at the schools were informed about possible further diagnosis and intervention in particular cases.

#### Description of the intervention

The study builds on a disordered eating prevention program that was developed in a previous project and whose efficacy was already evaluated [88]. The intervention was based on the social cognitive model, the media literacy educational approach and cognitive dissonance theory. The intervention program’s approach can be classified in terms of socio-cultural resistance skills [47]. Preventive programs based on the social cognitive model are focused on specific factors that may be involved in the development of the problem. The environment is a central element because it provides reference models. Consequently, the aim is to provide new models for promoting healthy behaviors and counteracting the effects of other models less healthy than those present in the social environment [91,94]. Another fundamental element is the generation of self-efficacy beliefs to promote behavior change [95]. The educational approach called Media Literacy provides a reference framework for teaching children and young people to decode and interpret media messages, and for developing capabilities and skills of critical analysis that allow them to reduce the influence of such messages [96,97]. Media literacy is the commonest approach used by the most effective classroom-based body image programs [47]. Finally, dissonance theory, from social psychology, states that inconsistent cognitions create psychological discomfort that motivates people to alter their cognitions so as to produce greater consistency [98]. The most effective targeted prevention programs in this field are dissonance-based interventions [99].

The intervention has two components. The first component, called Nutrition, consists of two 60-minute sessions. The main objective of this component is to increase knowledge about basic concepts in nutrition and food and correct false beliefs about food. This knowledge is transmitted from a positive approach: it focuses on what students can enjoy rather than what they should avoid [100,101]. In the second session the practical and relevant aspects of food are discussed through the analysis of four menus (one balanced and three unbalanced in carbohydrates, proteins and fats). The second component, called Media Literacy, consists of four 60-minute sessions plus two 60-minute activity sessions. The content included in each session is show in Table 2. The presentations and group activities of this component are aimed at: (1) questioning the thinness ideal as a unique, healthy and objective ideal, through an inductive approach; (2) showing how to create a media message; and (3) developing critical thinking and active behaviors toward messages conveyed by the media. This component is based on the “Action learning” model for developing media literacy skills [96,97], and through the activities it is also intended to generate cognitive dissonance in relation to the current Beauty Ideal. Dissonance is maximized when participants feel that they voluntarily assume the counter-attitudinal stance, because otherwise they attribute their inconsistent behavior to the demands of the situation, and little attitudinal change results [98]. Therefore, the activities must be treated as voluntary, never as mandatory.

**Table 2:** Content and sequence of each of the Components in the Preventive Program

**Nutrition**	**Media Literacy**
*First session*	*Third session*
1. Eating and nutrition^1^	1. Feminine beauty ideal^1^
• Balanced eating concept	• Beauty throughout history
• Concepts of eating and nutrition	• Recent changes in the criteria of beauty
• Nutrients	• Beauty in different cultures
• Food pyramid and foods	• Thinness in Western culture today
• The importance of water	*Fourth and Fifth sessions*
	2. The feminine beauty ideal in the media^1^
	• Analysis of advertising messages and transmission of values
	• What advertising hides from us
	• The comparison trap
	• Introduction of the first activity
	*Sixth session*
	3. Activity 1: advertising analysis
	• Guided critical analysis of an advertisement
	• Responding to a 10-question media literacy-based script
*Second session*	*Seventh session*
• Analysis of menus (one balanced and three unbalanced)	4. How to deal with media messages^1^
	• Groups complete and discuss the work done in Activity 1
	• “You can do something”: How to develop active attitudes and behavior
	• Introduction of the second activity
	*Eighth session*
	5. Activity 2: complaint letters to the media
	• Preparing and writing a complaint letter
	• Giving the letter to one’s tutor^2^
Booster session	
• Summary of the main ideas transmitted in the *Media Literacy* component	
• Looking back at Activity 2: complaint letters to the media	
• Summary of the procedure for sending the letters and the main results:	
■ Number of companies to which letters were sent	
■ Most widely denounced advertisements	
■ Number of responses from the companies	
■ Summary of the main ideas reported	
■ Summary of the main ideas contained in the companies’ response letters	
■ An answer model: Response letter from Philips	

^1^Power Point presentation; ^2^The researchers collected and sent the letters to advertisers.

Nine months after completion of the intervention there was a 60-minute booster session. This session aimed to refresh memories of the main ideas conveyed in the Media Literacy component and to look at the companies’ responses to the letters of complaint written by the participants in one of the activities of the program, with a view to strengthening their activist behavior and increasing their self-efficacy beliefs (see Table 2). Duration of the program is one session per week for eight weeks plus one booster session. All sessions took place during the scheduled weekly tutor group sessions at each school.

The intervention was presented as a project focused on healthy eating habits and the issues of body image and the feminine Beauty Ideal. It was not explicitly presented as a disordered eating prevention program, as this covert approach has been found to yield better results [45,102]. The format is multimedia and interactive.

Finally, fidelity in the administration of the program was assessed to ensure that different community providers administer key elements of the program in a full and correct manner. To check the fidelity the researchers contacted those responsible at each school and checked that all scheduled sessions had been carried out.

### Measurements

#### Biographical data questionnaire

In order to be able to define more precisely the socio-demographic characteristics of the sample, the MABIC protocol included Hollingshead’s (1975) Four Factor Index of Social Status (FFISS), together with questions about the origin of the parent population and date of birth.

#### Weight status

Weight and height were measured in situ by trained research staff in a private room near the protocol administration classroom. Height was measured to the nearest millimeter using portable stadiometers SECA-214 (20–207 cm; accuracy range of 0.1 cm), and body weight was assessed to the nearest 0.1 kg using digital scales SECA-872 (0–200 kg; accuracy range .1 kg; precision ± 0.15%). For taking the measures, participants were required to stand up, without shoes, wearing light clothing and without any personal objects (such as watches, bracelets or mobile phone). Weight values were subsequently corrected by subtracting .9 kg from the boys and .7 kg from the girls, which were average values estimated after weighing several sets of clothes similar to those worn at the time of the assessment. BMI (kg/m^2^) was calculated and used to obtain the weight status categories (underweight, normal weight, overweight and obesity), using international cut-off points for participants’ age [103,104]. Weight and height measures were taken only at pretest and at the 1-year follow-up.

#### Sociocultural Attitudes Toward Appearance Questionnaire–3 (SATAQ-3)

The SATAQ-3 [105] and its previous versions is the instrument most widely used today for assessing sociocultural pressures and the internalization of the beauty ideal (M of MABIC). Moreover, it is commonly used worldwide to determine changes in internalization in this prevention field [47]. It is a 30-item scale with four subscales. Two of these subscales are based on different beauty ideal internalization factors. The first is “Internalization-General” (SATAQ-IG), with nine items (e.g., “I try to look like the people on TV”), and assesses general media influence related to TV, magazines, and movies. The second is “Internalization-Athlete” (SATAQ-IA), with five items (e.g., “I try to look like sports athletes”), and assesses the internalization of athletic and sports models. The other two subscales are “Information” (SATAQ-I), with nine items (e.g., “TV programs are an important source of information about fashion and “being attractive”), which assesses how far it is acknowledged that various media are considered important sources of information about appearance, and “Pressures” (SATAQ-P), with seven items (e.g., “I’ve felt pressure from TV or magazines to diet”), which assesses subjective feelings of pressure from exposure to media images and messages to modify one’s appearance. The response format is a Likert-type scale ranging from 1 (completely disagree) to 5 (completely agree). We used the Spanish version of the SATAQ-3, validated with adolescent boys and girls by our research team [106], and in which Item 20 from the “Internalization Athlete” subscale was excluded. In the Spanish validation study, the internal factor structure was evaluated by means of exploratory structural equation modeling (ESEM). The results supported the four-factor solution of the original version, and revealed a good fit, excluding Item 20 and with correlated uniqueness between reverse-keyed items (ESEM / CFI = 0.96; TLI = 0.94; RMSA = 0.047). The version showed invariance by sex and grade, providing substantial endorsement for the possibility of using the same version of the questionnaire with both boys and girls and throughout a large portion of adolescence. The differences between scores of different groups were in the expected direction, and supported the validity of the Spanish version. The reliability estimates were .840, .775, .859 and .775 for the scales SATAQ-IG, SATAQ-IA, SATAQ-P and SATAQ-I, respectively. All of these values can be considered as satisfactory.

#### Children Eating Attitudes Test (ChEAT)

The ChEAT [107] is a well-established scale including 26 items (e.g., “I feel guilty after eating”) designed to assess maladaptive or problematic eating attitudes and behaviors among children and adolescents (A of MABIC). It is also one of the most commonly used scales worldwide to determine changes in disordered eating in this prevention field [47]. We used the validated Spanish version [108], whose internal consistency is .73. In this version, 6 items of the original 26 were removed due to the requirements of the institution that authorized the carrying out of the study. In our research we maintained the wording of the 20 items included in the version and added the missing 6. The Spanish version suggests using a conservative cut-off point below 17 to identify possible risk cases, given that the specificity was very good (between 94 and 100%) but the sensitivity very low (between 2 and 8%). Internal consistency was 0.71. We are aware of the existence of a more recent Spanish version, carried out with a large sample and with better indices of sensitivity and specificity [109], but this version was not available at the time of the baseline assessment.

#### Perception of Teasing Scale (POTS)

The POTS [110] is also the most widely used scale for determining changes in appearance-related teasing (B of MABIC) in this preventive field [47]. It consists of 11 items and assesses one’s history of being teased about weight and abilities/competencies. The original scale yields a six-item Weight-Related Teasing subscale (POTS-WT) (e.g., “People made jokes about you being too heavy”) and a five-item Competency-Related Teasing subscale (POTS-CT) (e.g., “People said you acted dumb”). Each item comprises two parts, the first of which is aimed at the assessment of teasing frequency, and answered on a five-point scale ranging from 1 (never) to 5 (very often); the second part of each item deals with the effect of the teasing, and requires a response only if the participant gives an answer other than “never” to the first part. This second part also has five response categories, from 1 (not upset) to 5 (very upset). Total score on each item is the sum of the score on the two parts. The 9-item Spanish version of the POTS (POTS-S), also validated by our research team, was used for this study [111]. The two-subscale structure was supported by means of confirmatory factorial analysis, and revealed a good fit, excluding Item 1 and 6 from the POTS-WT subscale (CFI = 0.97; TLI = 0.96; RMSA = 0.038). Internal consistency was .86 and .76 for the POTS-S-WT and POTS-S-CT subscales, respectively. Test-retest reliability was .85 and .65. The POTS-S showed good correlation indexes with other, related variables. As was the case with the Spanish version of the SATAQ-3, the POTS-S version showed invariance by sex and grade, giving support for its use with both boys and girls throughout a large portion of adolescence.

#### Eating Disorders Inventory-3 (EDI-3)

We used the “Body Dissatisfaction” subscale (EDI-BD) and “Drive for Thinness” subscale (EDI-DT) from the EDI-3 [112] in its Spanish version [113]. These two instruments are those most widely used for determining changes in body dissatisfaction (IC of MABIC) and drive for thinness in disordered eating universal prevention research worldwide [47]. The EDI-BD scale contains 10 items that measure satisfaction with specific body sites, such as the waist, thighs and buttocks. Response options ranged from 1 (never) to 6 (always). The validated Spanish version presents an internal consistency of .87 to .88 for girls between the ages of 10 and 18. The EDI-DT is a seven-item scale that measures restrictive tendencies, including excessive concern with dieting, weight concerns, and pursuit of thinness. Response options ranged from 1 (never) to 6 (always). The validated Spanish version has an internal consistency of .85 to .89 for girls aged 10 to 18.

#### Positive And Negative Affect Scale (PANAS)

We used the 10-item “Negative Affect Scale” (PANAS-N) of the PANAS [114] in its validated Spanish version for children and adolescents [115]. Respondents are required to respond in accordance with how they normally feel and/or behave (e.g., guilty, shameful, nervous) on a scale with three options; “Never”, “Sometimes”, and “Often”. The internal consistency was .75 to .74 for girls and boys.

#### Rosenberg Self-Esteem Scale (RSES)

The RSES [116] was used to measure adolescent self-esteem. Once again, it is among those most widely used for measuring changes in self-esteem in disordered eating universal prevention research worldwide [47]. The 10-item scale has a 4-point Likert-type response scale ranging from 1 (strongly disagree) to 4 (strongly agree). We used the Spanish validation [117], which has an internal consistency of .85 to .88.

#### SCOFF

The Spanish version of the SCOFF [118], the SCOFF-c [119], was used as screening tool for possible cases of eating disorders. It consists of five questions that represent the current consensus on the main features of eating disorders. It is one of the most prestigious eating disorders screening questionnaires currently in use, given both its ease of application and its good psychometric properties. Each Item is answered in a dichotomous way (Yes / No). In the Spanish version, scores ≥ 2 are considered to indicate risk for eating disorders, with a sensitivity of 73.08% and a specificity of 77.74%.

#### Ad hoc questionnaire for the evaluation of processes

Community providers were asked about the aspects of the training that helped them most in the administration of the program (the training received, the manual, the video-model, or others), and whether they had continued or intended to continue implementing the program. In addition, they were asked to indicate on a 10-point Likert-type scale the level of usefulness of the training received for administration of the program, the interest shown by the participants, the level of satisfaction with regard to their own administration, the degree of satisfaction with the management of the project by those responsible for it, and the degree of overall satisfaction with the project.

### Statistical analysis

Because adolescents within the same schools will likely display similar correlated values in several variables, in line with, among other factors, the teacher’s or peers’ influence, we shall analyze the data by means of Generalized Estimating Equations (GEE; [120,121]), which extends generalized linear models to accommodate dependent/correlated outcomes for clustered data (“School” in this study). GEE can be characterized as a population-averaged model (or marginal model), in the sense that it measures the expected (marginal) change in a response as the value of the covariate increases by one unit and, conceptually, is preferable for estimating the effect of cluster-level covariates such as intervention effects [122,123]. The GEE approach uses the generalized linear model to estimate more efficient and unbiased regression parameters relative to ordinary least squares regression, in part because it permits specification of a “working correlation matrix” that accounts for the form of within-subject correlation of responses for the dependent variables of many different distributions. This orientation focuses on correlated data arising from the relatedness of several individuals in the same cluster or panel (“School” in this study), rather than several “longitudinal” observations in the same individuals [124,125].

From this perspective, the data analysis strategy is based on defining “School” as a panel variable, and models are adjusted with the normal distribution (Gaussian family) and identity link-function and exchangeable working correlation matrix, and employing robust standard error. Because the outcome variable has to be considered at 3 points (baseline, post-test and 1-year follow-up), evaluation of the effects of the intervention will be conducted in two phases. First, we study the effect by gender on outcome at post-test taking outcome at baseline as a covariate in addition to group (control/intervention), BMI, age, socioeconomic status and origin of the parent population; subsequently, we will apply the same strategy on outcome at the 1-year follow-up. All analyses are performed with Stata 12/SE.

## Discussion

Schools are recognized as an appropriate setting for the prevention of a broad spectrum of eating and weight-related problems because they allow access to a large number of individuals and provide an opportunity to prevent these problems at every developmental stage [47,126,127]. In this context, teachers and other community providers should be those chiefly responsible for the administration of this type of preventive intervention [128,129]. However, data from a meta-analytic review [46] show that larger effects were found for programs delivered by experts or professional researchers than for those delivered by endogenous providers such as teachers. One of the main reasons for this result may be due to lack of training for the teachers who implement the interventions [130]. Very often, it is assumed that professionals such as teachers are qualified to implement programs for the prevention of eating disorders, overweight and obesity. However, several studies have indicated that knowledge among school staff and other community providers about nutrition, eating disorders, issues related to the mechanisms of weight control, obesity and the use of preventive techniques is poor or insufficient [129-134]. Because of this lack of knowledge, these professionals may unintentionally provide participants with inappropriate advice and/or misinformation about healthy weight control and display attitudes and behaviors that perpetuate the thinness ideal [127,130,132]. This is particularly noteworthy with regard to the causes of obesity, which are often attributed to individual behaviors that in turn could lead to the stigmatization of overweight children and adolescents [129,130,135,136]. Therefore, developing appropriate attitudes and knowledge in relation to eating disorders and obesity among teachers and other community providers through training programs is an essential component in the effectiveness of prevention programs.

To our knowledge, only three effectiveness trials, in addition to our own, have developed training programs aimed at teachers and other community providers that empower them to implement prevention programs in this field: The PriMa program [66-68], The Body Project [61] and the POPS program [69]. In particular, our training program includes all the elements identified by teachers as necessary for involvement in the administration of such interventions [128,137]: (1) in order to increase their motivation, information on the need to carry out this type of interventions was included when the training started: (2) updated information on the issues to be addressed in the program was incorporated; (3) participation was voluntary and intervention was implemented only by motivated community providers; (4) community providers had the support of their management teams and the relevant training was officially accredited; (5) useful materials for implementing the intervention were provided, such as the program manual, the video-model and informational documents about the most relevant content and techniques; finally, (6) we maintained constant supervision so as to be able to deal with any questions or problems arising and help them to feel supported.

As mentioned above, it will be vital to conduct effectiveness trials, which test whether interventions are effective when delivered by community providers under ecologically valid conditions, as opposed to tightly controlled research trials [58]. Very few studies in the disordered eating prevention field have evaluated whether a program administered by experts in an efficacy trial has similar effects when administered by community providers. The few effectiveness trials conducted to date have revealed that interventions which produced effects in well-controlled efficacy trials where research staff delivered the intervention often have smaller intervention effects under real-world conditions [59]. This was the case with the effectiveness trial of the Body Project program [61], in which the authors concluded that training and supervision of the community providers should be improved. As we have a previous efficacy trial of the same prevention program, our results can be compared and new contributions will be made in relation to this transition from efficacy to effectiveness [54].

Our study incorporates some elements of the integrated approach to the prevention of eating disorders and obesity. On the one hand, in terms of the content of the program itself, it incorporates a component entirely devoted to promoting a balanced diet with a positive approach to nutrition education. On the other hand, the training of community providers implemented entirely from this approach. And finally, as regards the variables assessed, it incorporates a broad set of relevant variables for the entire spectrum of eating and weight-related problems.

The current protocol study has a number of strengths. First, to our knowledge, our study is the first effectiveness trial in disordered eating universal prevention research whose efficacy has been previously established. Its results could provide relevant data contributing to the transition from efficacy to effectiveness in this prevention field. Second, the training program for the community providers constitutes a considerable improvement on other training programs conducted to date in this field. Third, it incorporates several elements of the integrated approach to the prevention of both obesity and eating disorders. Fourth, the measures employed to assess effectiveness are among those most widely used to determine changes in the disordered eating and body image prevention field worldwide, and have been validated for Spanish population. Finally, height and weight were measured *in situ*, in contrast to the case of many studies with adolescents that rely on self-reported height and weight data.

A series of limitations should also be considered in relation to this study protocol. In contrast to our efficacy trial, in the effectiveness trial the randomization of groups was not possible. This is a controversial issue. The effects of randomized and non-randomized studies are very well correlated, but non-randomized studies tend to show larger treatment effects [138]. However, in some fields of prevention, such as that of obesity prevention, differences between the effects found in randomized and non-randomized trials have not been significant [139]. For some researchers, for a program to be found effective, it must also meet all standards for efficacy, including randomization [51]. But sometimes randomization is impossible under real-world conditions [54]. Randomization, or even being able to have a control group, is difficult to attain due to requirements of the participating entities [140]. That was the case in our study, and randomization was not possible. The use as a control group of participants from a neighboring area with characteristics similar to those of the intervention group, as in our case, is an option already used in other effectiveness trials in prevention [141]. This option can help avoid a “spillover” effect, and possible demographic differences could be controlled with strategies in place to match samples for demographic characteristics, or through covariate analyses [47]. It should be noted that our results will be adjusted in line with the main variables, and adjusted results from non-randomized experiments can approximate to results from randomized experiments [142]. Finally, the sample in this effectiveness trial is not representative of the entire population of Spanish adolescent girls and boys, and the results we obtain must be treated with caution before making generalizations for the entire population.

Finally, the Society for Prevention Research has designed Standards to determine which interventions are efficacious and effective. An effective intervention under these Standards will not only meet all standards for efficacious interventions, but will also: (1) make manuals, appropriate training, and technical support available to allow third parties to adopt and implement the intervention; (2) have been evaluated under real-world conditions in studies that included sound measurement of the level of implementation and engagement of the target audience (in both the intervention and control conditions); (3) have indicated the practical importance of intervention outcome effects; and (4) have clearly demonstrated to whom intervention findings can be generalized. Our prevention program has met the Standards for efficacy in our efficacy trial [88]. Our ongoing effectiveness trial already meets the first two Standards for effective interventions. The other two are related to the final results for which we are waiting. In conclusion, this study will provide new evidence to improve and enhance disordered eating prevention programs.

### Trial status

The design of the MABIC training program and the training of the community providers have already been completed. Intervention has been implemented and pre, post and 1-year follow-up have been carried out in both intervention and control groups. In May 2013 we received the final responses from the community providers about process assessment. Baseline and post-test assessment data were entered in the database, and 1-year follow-up data were entered in June 2013. Neither data cleaning nor any analysis had begun at the time of submission.

## Abbreviations

APA: American Psychological Association; BMI: Body Mass Index; CSPT: Acronym in Spanish for Parc Taulí Health Corporation; GAP: Grant-aided private; FIP: Acronym in Spanish for Institute of Psychology Foundation; MABIC: Acronym in Spanish for four shared risk factors of eating disorders and obesity with empirical support: “M” for media (“Medios de comunicación” in Spanish); “A” for disordered eating (e.g. dieting) (“Alimentación alterada” in Spanish), “B” for weight-related teasing, (“Burlas relacionadas con el peso” in Spanish) and “IC” for body dissatisfaction (“Insatisfacción Corporal” in Spanish).

## Competing interests

The authors declare that they have no competing interests.

## Authors’ contributions

DSC managed the MABIC project and supervised all phases of the study protocol. DSC and GLG designed and implemented the training program. DSC, GLG, JF, JRB, JP, MQ and ET participated in data collection in the pre, post and 1-year follow-up assessments. MP and MQ helped to recruit the health community providers and the participating schools. MP helped to organize the training program. JF and JRB participated in the design of both the study and data analysis strategy. DSC, GLG and JF drafted the manuscript. All authors read, revised and approved the final manuscript.

## Pre-publication history

The pre-publication history for this paper can be accessed here:

http://www.biomedcentral.com/1471-2458/13/955/prepub
